# Cold Bonding Method for Metallic Powder Coatings

**DOI:** 10.3390/ma11112086

**Published:** 2018-10-25

**Authors:** Wei Liu, Jing Fu, Haiping Zhang, Yuanyuan Shao, Hui Zhang, Jesse Zhu

**Affiliations:** 1 School of Chemical Engineering and Technology, Tianjin University, Tianjin 300350, China; wliu364@uwo.ca (W.L.); hpzhang@tju.edu.cn (H.Z.); yshao@tju.edu.cn (Y.S.); 2 Department of Chemical and Biochemical Engineering, Western University, London, KY N6A 5B9, Canada; jfu33@uwo.ca (J.F.); jzhu@uwo.ca (J.Z.)

**Keywords:** powder coating, metallic flakes, bonding

## Abstract

An efficient and simple method for preparing bonded metallic powder coating is in high demand in the paint manufacturing and application industries. The bonding purpose is to keep the mass percentage of metallic pigment consistent between the original and recycled coating powder, which aims at solving the problem of recyclability. One possible method capable of realizing this goal is using the binder to cohere metallic pigment with base particles through a cold bonding method. Through this approach, the pre-curing and high-reject-rate problems generally present in thermal bonding can be completely eliminated. In this paper, polyacrylic acid (PAA) and polyvinyl alcohol (PVA) are applied as binders for the bonding process. At various dosages of liquid binder and D.I. water, bonded samples with different bonding effect were prepared. Finally, a good bonding quality with the lowest variance between the mass concentrations of Al flakes in the original powder (before spray) and deposited powder (after spray) 2.94% with PAA as a binder and 0.46% with PVA as a binder was achieved. These results manifest that the cold bonding method is a green and simple approach for preparing the metallic powder coating.

## 1. Introduction

Metallic powder coating, which is a powder coating incorporated with metallic pigment(s), has been gaining increased market share in recent years [1,2,3,4,5]. Powder coatings are dry paints formulated with resins, pigments, and additives, which are similar to the solvent borne paints with the exception of no solvent present; while metallic pigments, such as aluminum, copper, nickel and zinc, are introduced for providing better aesthetic appearance and protection. Metallic powder coatings are usually applied on premium products, for instance, automotive, computer and high-end appliance parts etc. [6,7,8,9,10]. Their final films exhibit extra shine and deepness to the color by introducing a metallic effect. Initially, all metallic powder coatings were non-bonded, which means that a powder base coat was prepared and then metal flakes were dry-blended into the powder to create a metallic effect [11,12,13]. There is no adhesion between metallic flakes and base particles. This method is simple, but the product is unstable, due to the poor recyclability caused by non-bonding between the two. Recyclability means that over-sprayed powders (about 10 to 40%), which failed to deposit onto a substrate at first spray, can be re-sprayed after being recycled by a powder collecting system [14,15]. This is one of the biggest advantages of powder coating [14]. The sharp distinctions between base particle and metallic flake, such as shape, surface conductivity, volume and mass, result in a difference in the concentrations of the metallic pigment in the final film and the original coating powder when using the electrostatic spraying process, which is the main spraying method in the powder coating industry due to its ability to realize full-automatic painting with thick films (50–300 μm) on a large scale [16,17,18]. This difference of course leads to the inconsistency of the metallic pigment concentrations between the over-sprayed powder (the part that fails to deposit onto the work piece) and the original coating powder, making the over-sprayed non-recyclable. Additionally, the electrostatic spraying makes the metal flakes “bunch” together due to electrostatic force, and therefore causes a non-uniform shininess over the final coating film. In addition, there are some other drawbacks with non-bonded product, such as metallic flakes accumulating on the head of the spray gun, which has a high risk of discharging and burning [19]. On the contrary, a fully bonded product can overcome all of these problems.

Melt-extrusion and thermal bonding are the two methods used to produce bonded metallic powder coating in current industries [20,21]. The first approach follows the same production process of the conventional plain powder, except that the pigments contain strip-shaped metallic flakes with special surface treatment. All these ingredients (pigments, resins, additives, and fillers) are well-mixed, melt-extruded, and then ground into powders, which are the final bonded product. Apparently, metallic flakes and base particles have been well bonded with each other after the melting process, which makes this kind of bonded product with excellent operation stability. However, the mixing and shearing in the extruder at a high temperature are more likely to change the pigment particle size and ruin the metal surface effect [3,22]. Therefore, the melt-extrusion method did not find wide application in the powder coating industry for the main-stream sheet-shaped metallic pigments. In the thermal bonding method, metallic flakes and coating powders are heated in a chamber by high-speed stirring until the temperature precisely reaches the softening point (glass transition temperature) of the coating powder [23]. Then, the sticky surface of the base particles glue up the metallic flakes. This approach not only solves the problems in the non-bonding and melt-extrusion methods, but also gives the product a shiny and uniform final appearance. With this method, the temperature control of the blending process is critical [23,24]. If it is under the softening temperature, there will be no bonding between metallic flakes and base particles; and once it exceeds this point by a couple of degrees, particles of the powder are easily bonded with each other (mis-bonding) and may even cause pre-curing. This requires bonding machines that are competent for precisely controlling the inner temperature. Moreover, in industrial applications, the temperature is generally set a little higher than the softening point to ensure well-bonding, which would increase the possibility of mis-bonding base particles and pre-curing of coating powders [25]. Therefore, in order to ensure the quality of metallic powder coating, post-treatments (such as grinding and sieving) are necessary. These issues make the bonding process complicated, expensive, and low-efficient, which limits the application level and field of the metallic powder coatings.

Thus, seeking an efficient, simple-operation and cost-saving bonding method is the objective of this paper, which is highly demanded for expanding metallic powder coating to wider applications, such as coatings for bikes, regular appliances, and decorative building materials. To achieve this objective, a novel cold bonding method through polymers by high-speed stirring is proposed. Polymers that are water soluble, adhesive as well as compatible with resin (i.e., polyester in this paper) can be the candidate binders. Based on previous work [26,27], two types of candidate binder polymers were used to bond aluminum flake and a base coating particle at room temperature. In addition, Al flake was used as metallic pigment in all experiments, because more than 90% of metallic pigments in this field are Al flakes. These binders work like glues between the Al flakes and coating particles and bind them by adhesive and cohesive forces. After a detailed investigation on the dependence of bonding quality on different binder-water formulas, the optimal formula was determined, and a simple and economic cold bonding method was developed.

## 2. Experimental

### 2.1. Materials

The following materials were employed in this study: Aluminum flakes (SILBERCOTE PC 3101X, with inorganically treated surface) from Silberline Manufacturing Co., Inc. (Tamaqua, PA, USA) was used as metallic pigments; Water soluble polyacrylic acid (PAA A-725, (CH_2_CH)_n_COOH) from Macklin Biochemical Co., Ltd. (Shanghai, China) and polyvinyl alcohol (PVA 1788, (C_2_H_4_O)_n_) from Sanwei Co., Inc. (Shanxi, China) was chosen as two binders; High gloss polyester clear powder coating (D50 = 44 μm, with 7 wt.% triglycidyl trimeric isocyanate as crossing linking catalyst, 9910-01289) from TCI powder coating Co. (Ellaville, GA, USA) worked as base powder coating; Al panel (A-2-3.5) from Q-Lab Co. (Westlake, OH, USA) and contrast panels (T12G, 76 mm × 132mm ) from Leneta Co. (Mahwah, NJ, USA) were utilized as substrates.

### 2.2. Equipment

Mixer (CBG100SC) from Applica Consumer Products, Inc. (Miramar, FL, USA) was performed in Step 3 of the bonding process; Electrostatic powder coating system (Surecoat Manual) from Nordson Co., Ltd. (Westlake, OH, USA) was used for the coating substrate.

A laser particle size analyzer (BT-2000B) from Bettersize Instruments Ltd. (Liaoning, China) was utilized to analyze the size distributions of the powder samples. A Scanning Electron Microscope (SEM) (S-4800) from Hitachi Limited (Tokyo, Japan) was employed to observe the bonding situation between Al flake and coating particle. The thickness of the final films was measured by a thickness meter (Positector 6000) from Defeisko Co. (Ogdensburg, NY, USA) and the final surfaces were characterized by an optical microscope (OT4975) from Mitutoyo Inc. (Kawasaki, Japan).

### 2.3. Bonding Method and Characterization of Metallic Powder Coating

**Figure 1:**
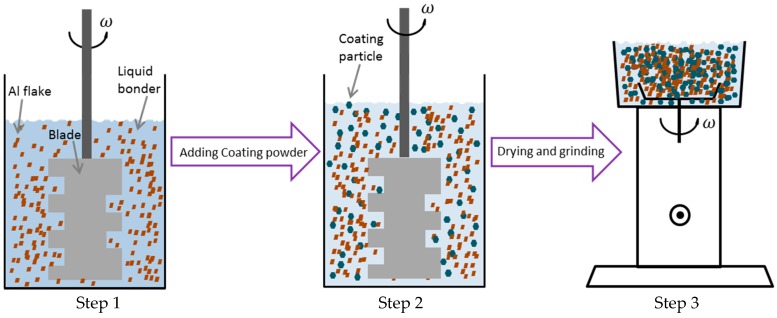
The process of cold bonding method.

The flow chart of the proposed cold bonding method for the metallic powder coating is shown in Figure 1. It started with the dissolving of the liquid binder (PAA or PVA) by D.I. water. Afterwards, a mixing process of the Al flakes and the binder solution proceeded, as depicted in step 1. After mechanically stirring (60 rpm) this mixture for 30 min, 15 g polyester clear coat (which does not contain fillers and pigments) was added and the mixture was stirred for another 30 min (step 2). In step 3, the half-wetted mixture was fed into a grinder. It was ground in the chamber through high-speed stirring for 20 s and dried up in air for 24 h; the final product was then collected.

The non-bonded or obtained bonded coating powders were sprayed onto one side of the Al panels by an electrostatic coating powder system. Due to the separation between Al flake and base particle is quite noticeable under extremely low or high spray voltages (−30 or −90 kV), these bonded products were electrostatically sprayed onto an Al panel (2 inch × 5 inch) at −30 kV, −30 μA. The spraying distance is 30 cm and the powder flow rate is approximately 50 g/min.

The bonded samples prepared by cold bonding method were first analyzed by an ash test [28], which aims at revealing the mass percentages of Al flakes in the original powder (before spray) and deposited powder (after spray). The deposited powders on the Al panel were scratched off into a crucible for the ash test. The polyester clear powder coating will be burned into CO_2_, H_2_O, other gases and trace amounts of ash residual (0.02 wt.%) at high temperature (530 °C), but the Al flake is almost not affected by the high temperature except receiving a nano-scale oxide film on the surface (~0.04 wt.%). With this approach, the content of Al in each powder sample can be obtained. The equation for calculating the mass percentage of Al flake in metallic powder coating is:

(1) $$\omega =\frac{M_{\mathrm{residual}}}{M_{0}}\times 100\%$$

Here, *ω* is the mass percentage, *M*_residual_ is the residual mass after the burning process. *M*_0_ is the mass of the sample before burning. Before spraying, the sample is its original powder coating; after spraying, the sample has the powder scratched off from the whole surface of the Al panel. As a result, the Al mass percentage in the original powder (before spray, *ω_orig_*) and deposited powder (after spray, *ω_dep_*) can be calculated by Equation (1). Obviously, a lower variance between *ω_orig_* and *ω_dep_* means a better bonding. Δω is defined by the following equation:

(2) $$\Delta \omega =\frac{\omega_{dep}-\;\omega_{orig}}{\omega_{orig}}\times 100\%$$

where, *ω_orig_* and *ω_dep_* are the mass percentages of Al flake in the powder coatings before and after spraying, respectively. It is clear that lower Δ*ω* represents a better bonding effect. To characterize the influence of the dosages of liqiud binder (*m_b_*) and water (*m_w_*) on the variance between *ω_orig_* and *ω_dep_*, different combinations of *m_b_* and *m_w_* were tested.

Based on the ash test, the best ones of these original powder samples were first gold coated and then characterized by SEM equipped with an energy-dispersive X-ray spectroscopy (EDS) analyzer. SEM images can directly display the bonding situation between base particle and Al flakes. Lastly, these samples were measured by size analyzer, which provides the size change information before and after the cold bonding process.

Non-bonded powder coating samples were prepared, sprayed and analyzed as a control group before the cold bonding experiments. The non-bonded (around 2 wt.% of Al flakes in 98 wt.% base powder) were made using simple dry blending with no binder and water.

## 3. Results and Discussion

### 3.1. Control Tests

**Figure 2:**
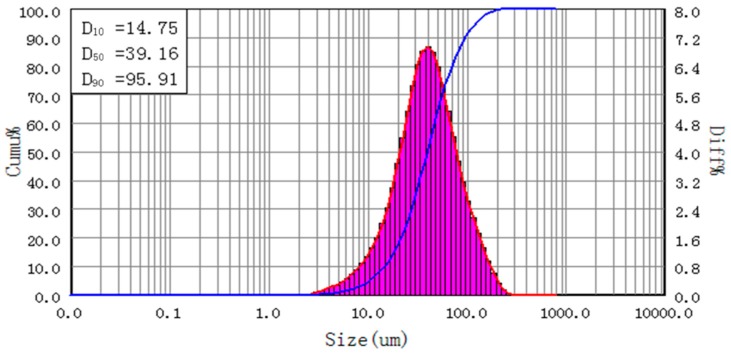
Size distribution of non-bonded sample.

The particle size distribution of control sample is depicted in Figure 2. It shows that the D_10_, D_50_ and D_90_ of the non-bonded powder are 14.75, 39.16 and 95.91 μm, respectively. The span calculated by (D_90_ − D_10_)/D_50_ is 2.07.

**Figure 3:**
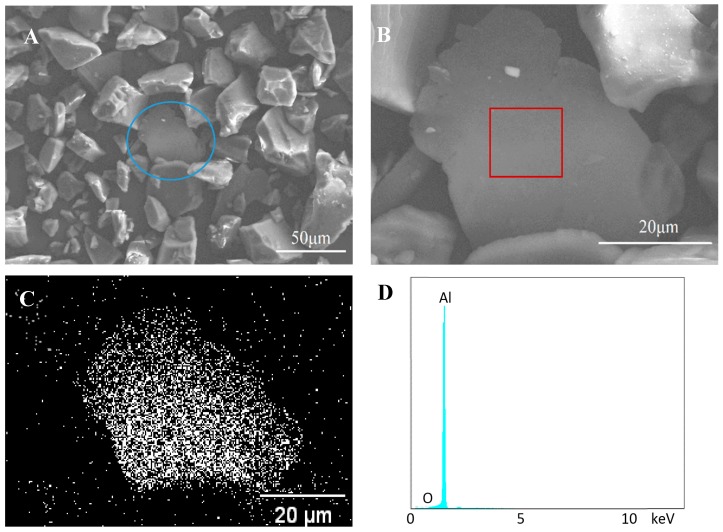
Scanning electron microscope (SEM) and energy-dispersive X-ray spectroscopy (EDS) images of control samples. (**A**,**B**) SEM images of non-bonded sample; (**C**) EDS scanning of aluminum element; (**D**) EDS spectrum of the rectangle area in (**B**).

Figure 3A displays some of Al flakes dispersed in the base particles, but no distinct bonding is observed between these flakes and particles. An Al flake without any particle on its surface is clearly seen at a high magnification in Figure 3B. This indicates there is no bonding between the flake and particle. The aluminum scanning result (Figure 3C) from EDS illustrates the plate in Figure 3B is an Al flake and those particles around the plate are coating powder. The energy-dispersive spectrum observed in Figure 3D further proves the flake is pure aluminum.

Table 1 presents the results of the ash test of Al mass percentage before and after spraying these samples, where each experiment was repeated three times. It can be seen that the mass percentages of Al flakes in the original non-bonded coating powders were 2.15 ± 0.02 wt.% (before spray), but the Al contents in the deposited powders increased to 5.08 ± 0.03 wt.% (after spray). The increase indicates that more Al flakes than needed were deposited onto the panel surface. In other words, the content of the Al flake in the over-sprayed coating powder was less than desired. The main reason for this result is that the Al flake can capture more electrons than base powder in a low electric field when spraying at −30 kV due to the lower dielectric constant of base particle.

### 3.2. Bonded by PAA

It was expected that both the dosages of binder and water would affect the bonding quality. In order to study the effects of binder and water dosages on bonding quality, different mass amounts of water and PAA resin were tested for the same mass of powder coating (15 g, contains 2.0 wt.% Al flakes). The results of the ash test are presented in Table 2. All the values of *ω_orig_* fluctuate around 2 wt.%, rather than remaining at ~2 wt.% (the Al content before cold bonding). The fluctuation is likely caused by the mass loss of Al flakes or base particle during the process of cold bonding. Because a high amount of binder may cause surface with orange peel (rough surface) or lower gloss and too much water will reduce the bonding effect, the dosages of binder and water were set to less than 0.060 g and 0.50 g, respectively. According to the control tests, the Al mass percentage in the deposited powders is 5.08 ± 0.03 wt.%. After being bonded by 0.01 g PAA, *ω_dep_* becomes even higher than in the control test, which implies that no effective bonding was formed and side effects may be generated during the bonding process. A possible reason for this result is that a dilute and thin PAA solution layer on Al flakes is not able to glue the base particle. But this layer increases the total electrons carried by the Al flake, which makes more Al flakes deposit onto the substrate. When the mass of PAA increases to 0.03 g, the Al contents in the deposited powder (*ω_dep_*) get closer to those of their corresponding virgin powders, except the one using 0.5g water. After using 0.06g PAA, the values of *ω_dep_* (2.25–2.10 wt.%) and *ω_orig_* (2.23–2.04 wt.%) are highly similar while the water dosage is less than 0.30 g. This indicates that the cold bonding method is able to gain a good bonding effect for the metallic powder coatings.

**Figure 4:**
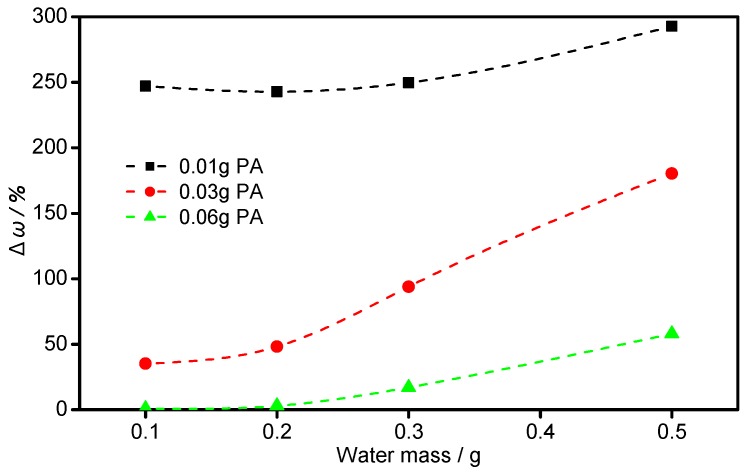
The relationship between Δ*ω* and the dosage of water and polyacrylic acid (PAA).

According to Mistry’s work, they used Gloss 20° and 60° to indirectly judge the bonding effect of the microwave bonding method [29]. In this work, the variance between *ω_orig_* and *ω_dep_* (Δ*ω*) was taken as direct and quantitative judgment of bonding efficiency. Figure 4 demonstrates the three series of mass content divisions before and after spray, Δ*ω*, which is plotted with respect to the water content. The samples with 0.01 g PAA have the highest Δ*ω*, exhibiting the worst bonding performance overall. As the amount of PAA increases, the Δ*ω* drastically decrease. The lowest Δ*ω* is obtained when the amount of PAA is 0.06 g. This is mainly because the bonding force increases with the dosage of the binder (PAA). In addition, the three curves also reveal that Δ*ω* can also be reduced by decreasing water content, which is due to the fact that the over-diluted binder tends to be removed from the Al flakes as a result of abration from the base particles. It is evident that in the tested PAA and water dosage ranges, a higher amount of PAA or lower amount of water is preferable for obtaining a lower Δ*ω*, namely a better bonding effect. In addition, the three curves display that the impact of PAA is greater than water on bonding. As to the optimum formula, the water dosage should not be kept too low, otherwise it can cause poor dispersion of the metallic pigment in the powder coating. Furthermore, too high a binder amount will have side effects on the final appearance. Therefore, the dosage of 0.06 g PAA with 0.2 g H_2_O is selected as the optimal formulation, whose Δ*ω* is 2.94%. In summary, almost equal Al mass percentages before and after spraying were obtained using the cold bonding method with proper PAA and water dosages, reflecting its great potential to achieve a desirable bonding performance.

**Figure 5:**
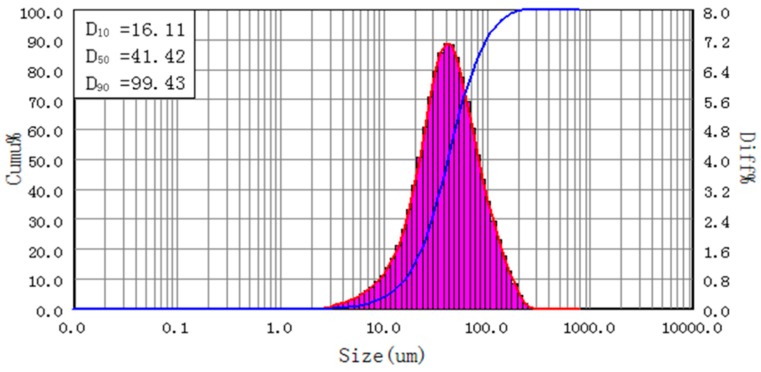
Size distribution of PAA bonded sample.

The sample prepared by 0.06 g PAA and 0.2 g H_2_O was further characterized. As shown in Figure 5, the D_10_, D_50_, and D_90_ of this sample are 16.11, 41.42, and 99.43 μm, respectively. Compared to the non-bonded one, both D_10_ and D_50_ increase by about 2 μm and D_90_ by 4 μm, which is reasonable because the base powder particles are bonded to Al flakes and formed larger particles.

**Figure 6:**
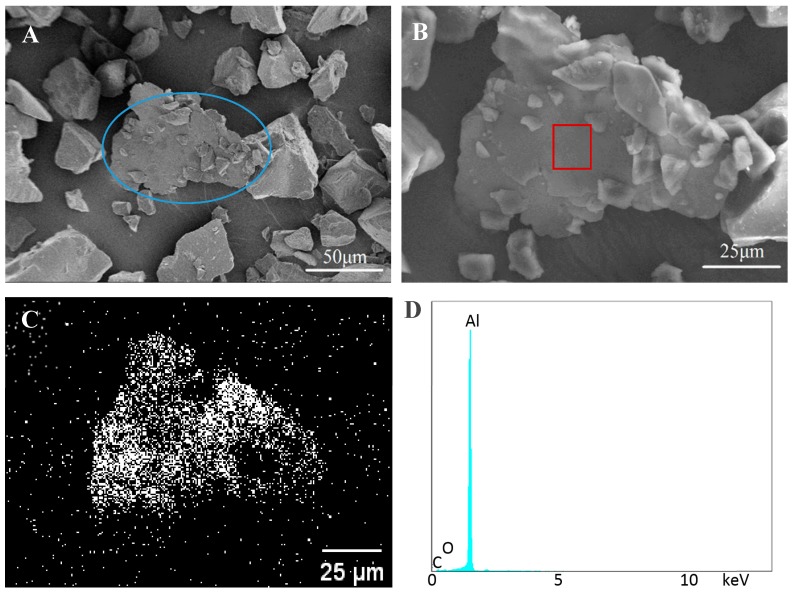
SEM and EDS images of PAA bonded samples. (**A**,**B**) SEM images of cold bonded sample by PAA; (**C**) EDS scanning of aluminum element; (**D**) EDS spectrum of the rectangle area in (**B**).

From the SEM images of Figure 6, it can be seen that many small (<20 μm) and medium size particles (~20 μm) were stuck on the surface of Al flakes, as exhibited in the circled area. This is because small or medium size particles have less mass so, the bond between the flake and particle is able to survive during mixing in the mixer. Gunde and co-authors also took SEM images as their judgment basis for thermal bonding [30], however, they had some mis-bounding between Al flakes, which is not found in this work. Additionally, there are some small particles bonded on the surface of the big particles as exhibited, which may also lead to size increase. This result also matches the conclusion of size distribution analysis. The EDS scanning for aluminum reveals that there are some dark areas (blockage of aluminum scanning) on the Al flake (Figure 6C), which coincide with the locations of the small particles seen from Figure 6B. This proves that the bonding of small particles on the Al flake surfaces occur. In addition, the energy-dispersive spectra (Figure 6D) of the rectangular area in Figure 6B confirms that this flake is comprised of aluminum. Small peaks of element of C and O are mainly caused by PAA. Above all, these images and element analysis verify the ash test result that bonding is indeed formed between Al flake and base particle.

### 3.3. Bonded by PVA

The previous results of PAA have proved the potential of cold bonding method for metallic powder coating. To verify the universality of the cold bonding method, another liquid binder, PVA, was investigated. Furthermore, the stickiness of the PVA solution is a little higher than PAA when at the same concentrations (0.5–5 wt.%) [31,32], so a lower amount of PVA was employed in this study. The dosages of binder and water are less than 0.016 g and 0.60 g, respectively.

Here, various combinations of PVA (0.008 to 0.016 g) and water (0.4 to 0.6 g) were studied, and the results are presented in Table 3. Table 3 shows the Al mass percentages of PVA bonded samples before (*ω_orig_*) and after (*ω_dep_*) spraying. All the values of *ω_orig_* are around 2 wt.%. When the mass of PVA and water are 0.016 g and 0.4 g, respectively, The *ω_dep_* is at its highest at 2.80 wt.%. After increasing the mass of water to 0.6 g, the Al content slightly decreases to 2.55 wt.%. This result implies that a high PVA dosage cannot produce the ideal bonding performance. When reducing the dosage of PVA solution to 0.008 g, the Al contents after spraying are lower than 2.29 wt.%, indicating that manifesting a lower amount of PVA (0.008 g) is better for providing a stronger bonding effect between the Al flakes and base particles. Overall, these data illustrate that PVA is also an efficient binder candidate for the cold bonding process of metallic powder coating.

**Figure 7:**
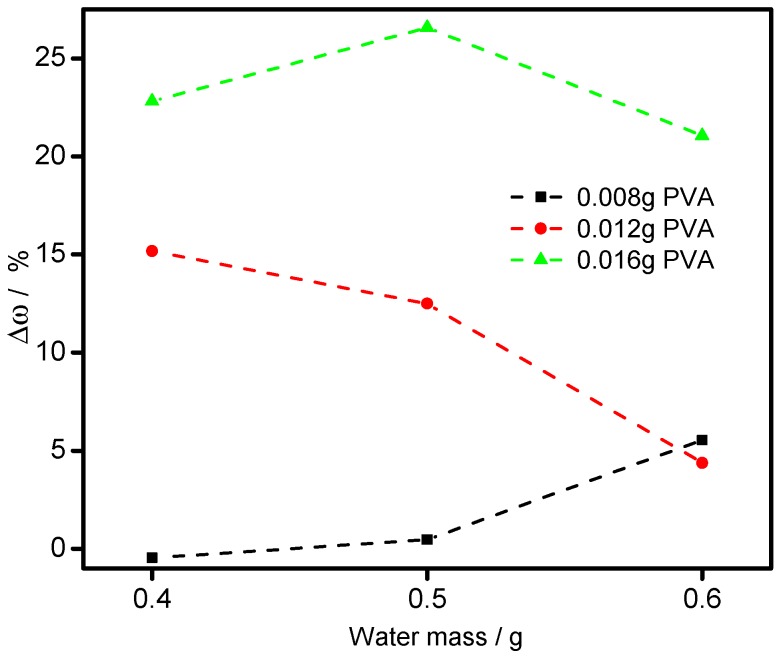
The relationship between Δ*ω* and the dosage of water and polyvinyl alcohol (PVA).

For further comprehending the relationship between *ω_dep_* and the dosages of PVA and water, the results were calculated by Equation (2), as illustrated in Figure 7. When bonded by 0.016 g PVA, all the variances are a little higher than 20%, which are much higher than other PVA dosages. This is mainly because the bonding force supported by 0.016 g PVA and 0.4 g water is too strong and causes mis-bonding between Al flakes in Step 1, which of course will hinder the bonding between Al flakes and base particles. When the amount of PVA is reduced to 0.012 g, the variance shows a decline during the increasing dosage of water (0.4 to 0.6 g). This means a better bonding effect was obtained after the diluting of PVA, which also implies that the mis-bonding between Al flakes in Step 1 was reduced. However, when bonded by 0.008 g PVA, the variance increases from −0.56 to 5.62% with increased amount of water from 0.4 to 0.6 g. This shows diluting by water will reduce the bonding effect, which is mainly due to the fact the low dosage of PVA (0.008 g) will not cause mis-bonding in Step 1, but the dilution will reduce the bonding between Al flakes and base particles in Step 2. In these samples, the lowest variance is 0.46%, which is gained by 0.008 g PVA and 0.5 g water.

In summary, the optimum formula (when Δ*ω* is 0.46%) in the PVA work is using 0.008 g PVA and 0.5 g water respectively. Compared to the optimal condition in the PAA section (when Δ*ω* is 2.94%), the lowest Δ*ω* of PVA is a little lower than the one from PAA. Overall, it is true that PVA is also a strong liquid binder for cold bonding method.

**Figure 8:**
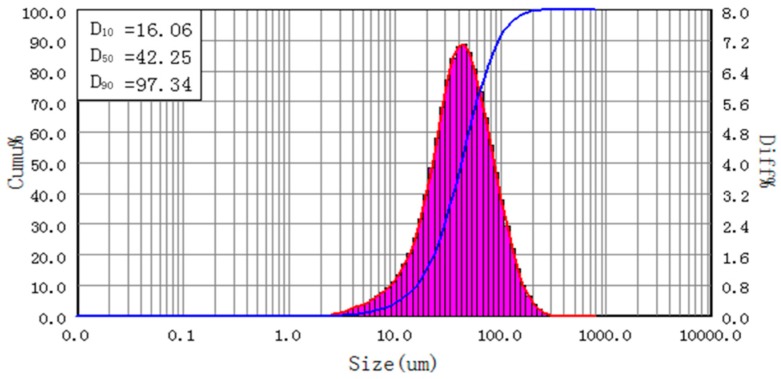
Particle size distribution of PVA bonded sample.

From the Δ*ω* analysis, the optimal sample is made by 0.008 g PVA and 0.5 g water. SEM and a size analyzer were applied to observe the bonding status of this bonded sample. The results are shown in Figure 8. The particle size of this sample is a little larger than that of the control, which reveals that a few small particles were glued on the surface of the larger ones.

**Figure 9:**
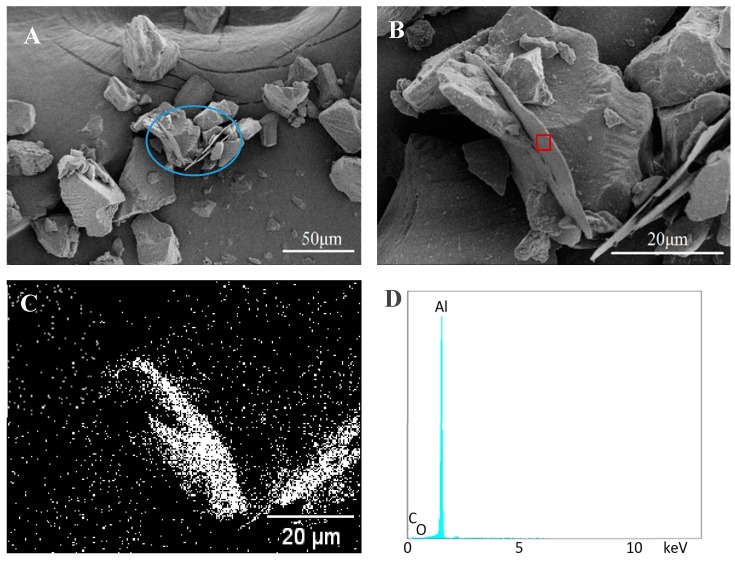
SEM and EDS images of bonded samples. (**A**,**B**) SEM images of cold bonded sample by PVA; (**C**) EDS scanning of aluminum element; (**D**) EDS spectrum of the rectangle area in (**B**).

Small particles bonding with large ones is also proved by the SEM images in Figure 9A. Although the particle size distribution of the bonded metallic coating powder has changed a little compared to the non-bonded sample, the influence on the final appearance is slight. There are several Al flakes bonded with base particles as exhibited in the circled area in Figure 9A. This area was magnified in Figure 9B, which clearly shows that both sides of Al flakes were well-bonded with base particles. In addition, the EDS scanning result shown in Figure 9C also reveals there are some base particles bonded on its surface. Figure 9D proves that the flake in Figure 9B is aluminum. The small peak in Figure 9D around 2 keV was caused by gold coating.

**Figure 10:**
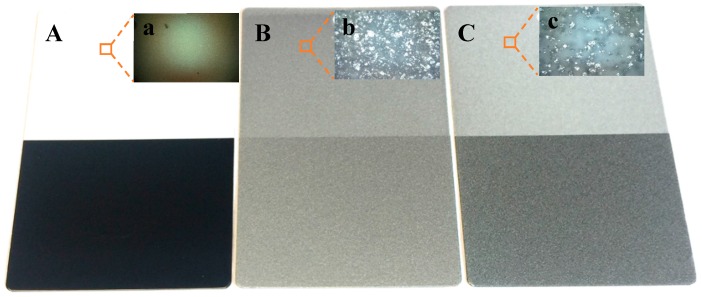
Surface comparisons between final films from various samples. (**A**) Clear coat, (**B**) Non-bonded sample, (**C**) Bonded sample.

In order to visually prove the bonding effect, some final films were prepared. Because the base coating powder is polyester clear coat, which is transparent after curing, special metal substrates (contrast panels, Leneta Company) with half black and half white surfaces were employed to create a better comparison after coating. After adding Al flakes as pigments, the colors of the substrate will be partially covered. It is understandable that more Al flakes in the final film means less original color of substrate will be shown. Based on the above results, the variance of Al content of PVA sample (0.46%) is a little lower than PAA (2.94%), even with less dosage comparing to PAA. Therefore, a final film, as shown in Figure 10C (thickness is 77.8 ± 6.1 μm), was obtained from a PVA bonded sample by spraying (at −30 kV) onto a metal substrate and then curing at 180 °C for 20 min. When without Al flake, the final film from the polyester clear coat is transparent as presented in Figure 10A (75.5 ± 6.7 μm). The final film from non-bonded samples with 2 wt.% Al flakes is shown in Figure 10B (81.0 ± 5.6 μm). It can be seen that the original color of substrate in Figure 10B is less exhibited than in Figure 10C, that is to say the Al content in final film from the non-bonded sample (Panel B) is higher than the PVA bonded sample (Panel C). An optic microscope was used to observe these final films as shown in the top-right insert images found in Figure 10. These microscopic pictures further confirm the visual observation. This conclusion is also in agreement with the ash test results, which shows the Al contents in deposited coatings from the non-bonding and PVA bonded samples are 5.08 and 2.18 wt.%, respectively. In addition, the Gloss and haze of panel C are 82.50 ± 0.76 and 21.6 ± 0.99 respectively, which are close to that of commercial one (83.03 ± 0.87 and 22.6 ± 1.02).

## 4. Conclusions

A cold bonding method employing a water soluble resin binder for fabricating metallic powder coating is proposed and tested. The results of the investigation suggest that: (a) the metallic powder coating can be well bonded by this cold method; (b) the bonding effect is highly dependent on the dosages of binders (PAA and PVA) and D.I. water; and (c) the proposed ash test method performs well in quantitatively characterizing the bonding effect of the bonded powder samples, while qualitative characterizing methods such as SEM and optical microscope observations, EDS scan, and visual observation can be used for further confirmation. By comparing the Al mass percentages in the original coating powder and deposited powder, the optimal formula in the tested ranges for PAA (the lowest Δ*ω* is 2.94%) is 0.3 g Al flakes with 0.060 g PAA and 0.20 g water; and for PVA (the lowest Δ*ω* is 0.46%) is 0.3 g Al flakes with 0.008 g PVA and 0.5 g water. In addition, the surface comparison suggests the bonded sample from this cold bonding method is able to have a similar surface quality with the one from a commercially bonded product. Therefore, cold bonding is an effective method to solve the bonding problems in the metallic powder coating. Further studies in this direction are expected to find other possible binders and establish a new approach for bonding the metallic powder coating.

## Figures and Tables

**Table 1:** Al content comparison of non-bonded powder (−30 kV).

Control Sample							
*ω_orig_*/wt.%				*ω_dep_*/wt.%			
1	2	3	Average	1	2	3	Average
2.14	2.17	2.15	2.15 ± 0.02	5.05	5.10	5.08	5.08 ± 0.03

**Table 2:** Al contents (wt.%) of the samples before and after spraying tests (−30 kV).

Content of Al	*ω_orig_*/wt.%			*ω_dep_*/wt.%		
H_2_O/g	**PAA/g**			**PAA/g**		
	0.010	0.030	0.060	0.010	0.030	0.060
0.10	2.19	2.19	2.23	7.60	2.96	2.25
0.20	2.10	2.18	2.04	7.20	3.23	2.10
0.30	1.99	1.98	2.22	6.96	3.84	2.60
0.50	2.06	2.19	2.14	8.09	6.14	3.38

**Table 3:** Al contents (wt.%) of the samples before and after spraying tests (−30 kV).

Content of Al	*ω_orig_*/wt.%			*ω_dep_*/wt.%		
H_2_O/g	**PVA/g**			**PVA/g**		
	0.010	0.030	0.060	0.010	0.030	0.060
0.40	2.12	1.91	2.28	2.11	2.20	2.80
0.50	2.17	2.08	2.07	2.18	2.34	2.62
0.60	2.17	1.83	2.11	2.29	1.91	2.55
